# Functions of MnO_x_ in NaCl Aqueous Solution for Artificial Photosynthesis

**DOI:** 10.1016/j.isci.2020.101540

**Published:** 2020-10-08

**Authors:** Sayuri Okunaka, Yugo Miseki, Kazuhiro Sayama

**Affiliations:** 1 Global Zero Emission Research Center (GZR), National Institute of Advanced Industrial Science and Technology (AIST), 1-1-1 Higashi, Tsukuba, Ibaraki. 305-8565, Japan

**Keywords:** Electrochemical Energy Conversion, Materials Characterization, Energy Materials

## Abstract

Photoelectrochemical water splitting has been intensively investigated as artificial photosynthesis technology to convert solar energy into chemical energy. The use of seawater and salted water has advantages for minimum environmental burden; however, the oxidation of Cl^−^ ion to hypochlorous acid (HClO), which has toxicity and heavy corrosiveness, should occur at the anode, along with the oxygen evolution. Here, O_2_ and HClO production in aqueous solution containing Cl^−^ on photoanodes modified with various metal oxides was investigated. The modification of MnO_x_ resulted in the promotion of the O_2_ evolution reaction (OER) specifically without HClO production over a wide range of conditions. The results will contribute not only to the practical application of artificial photosynthesis using salted water but also to the elucidation of substantial function of manganese as the element for OER center in natural photosynthesis.

## Highlights

• Selectivity of O_2_/HClO production in NaCl aq. sol. on photoanodes was investigated
• MnO_x_ modification promoted O_2_ evolution specifically without HClO production
• The results are useful for practical application of artificial photosynthesis
• A hypothesis about the evolution in natural photosynthesis was proposed

## Graphical Abstract

**Figure:**
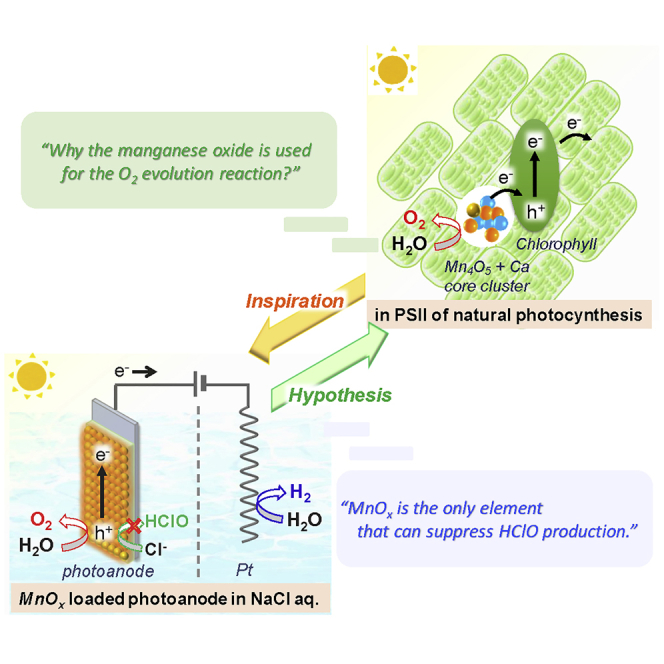

## Introduction

**Figure 1:**
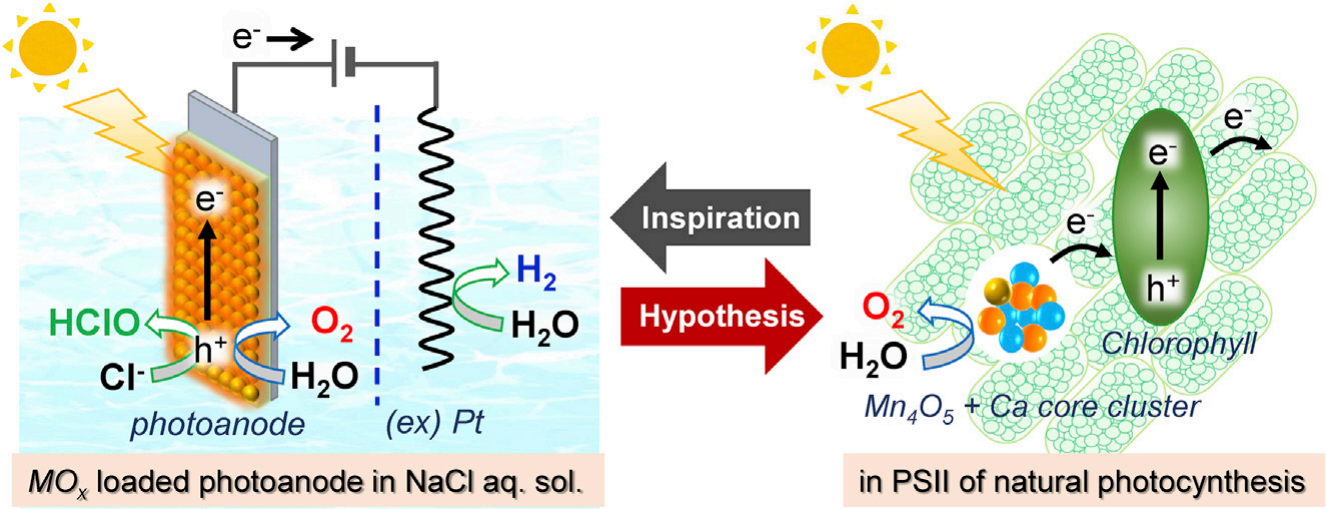
Reaction Images of the Artificial/Natural Photosynthesis Systems Reaction images of the artificial photosynthesis system using *MO*_*x*_*-*loaded BiVO_4_/WO_3_/FTO photoanode (left) and Mn_4_O_5_ + Ca core cluster for OER and chlorophyll in PSII of natural photosynthesis (right).

Various photoanodes composed of oxide semiconductors on conducting glass substrate have been widely studied for artificial photosynthesis technology with water oxidation into O_2_ under simulated solar light and low applied bias in aqueous electrolyte solution (Fujishima and Honda, 1972; Hisatomi et al., 2014; Kang et al., 2015; Sivula and van de Krol, 2016; Roger et al., 2017), whereby the efficiency was determined to be improved by loading with metal oxides (*MO*_*x*_) such as FeO_x_, NiO_x_, and CoO_x_ (Zhong et al., 2015; Chemelewski et al., 2014). The ability of O_2_ evolution reaction (OER) of *MO*_*x*_ has been further improved by modification with phosphate, borate, and carbonate ions (Kim and Choi, 2014; Bediako et al., 2012; Li et al., 2016). Moreover, the use of seawater and salted water for electrolyte solution has been investigated because of some significant advantages to minimize the environmental burden and provide cost reduction on electrochemical systems using photoanodes (Desilvestro and Gräetzel, 1987; Luo et al., 2011; Barczuk et al., 2013; Fukuzumi et al., 2017; Iguchi et al., 2018) as well as metal anodes (Bennet., 1980; El-Moneim et al., 2009; Vos et al., 2018; Ibrahim and Canan, 2015; Gidon et al., 2018). However, in seawater containing Cl^−^ anions, hypochlorous acid (HClO, neutral form of Cl_2_) production reaction (abbreviated as CPR) by Cl^−^ oxidation proceeds concurrently with the OER by water oxidation (Figure 1). Although HClO is a valuable chemical for disinfection and bleaching to some extent (Sayama, 2018), control of the selectivity and the suppression of CPR on photoanodes are important requirements for H_2_ production in large-scale water-splitting systems because of the toxicity and corrosiveness of HClO (Fujimura et al., 1999). The CPR via 2-electron reaction proceeds easily compared with the OER via 4-electron reaction, whereas the OER is more preferable to the CPR with respect to the redox potential (HClO/Cl^−^ = ca. +1.28 V and O_2_/H_2_O = +0.83 V versus SHE, pH = 7). There have been some reports that the CPR on metal anodes is effectively suppressed by the use of cation-exchange membranes or entirely coating the anode with MnO_x_ as Cl^−^-impermeable layers (El-Moneim et al., 2009; Vos et al., 2018; Balaji et al., 2009), and by buffered conditions to maintain an alkaline pH (Dionigi et al., 2016). However, it is more difficult to suppress the CPR in unbuffered NaCl solution because the anode is subjected to partially acidic conditions. Here, we systematically investigated the effect of loading various simple metal oxide species (*MO*_*x*_), including precious metals, on the selectivity toward HClO/O_2_ production from unbuffered NaCl aqueous solution over a BiVO_4_ photoanode, which is one of the most popular visible-light responsible photoanodes for efficient water splitting (Hong et al., 2011; Saito et al., 2012; Fuku et al., 2017), and found that MnO_x_ loading could specifically suppress the CPR on the photoanode under a wide range of conditions. Finally, we could advocate a new hypothesis why manganese is selected as the element for OER center of natural photosynthesis (Barber., 1998; Yano et al., 2006; Umena et al., 2011). (Figure 1)

## Results and Discussion

### Properties of *MO*_*x*_/BiVO_4_/WO_3_/FTO under Simulated Solar Light

**Figure 2:**
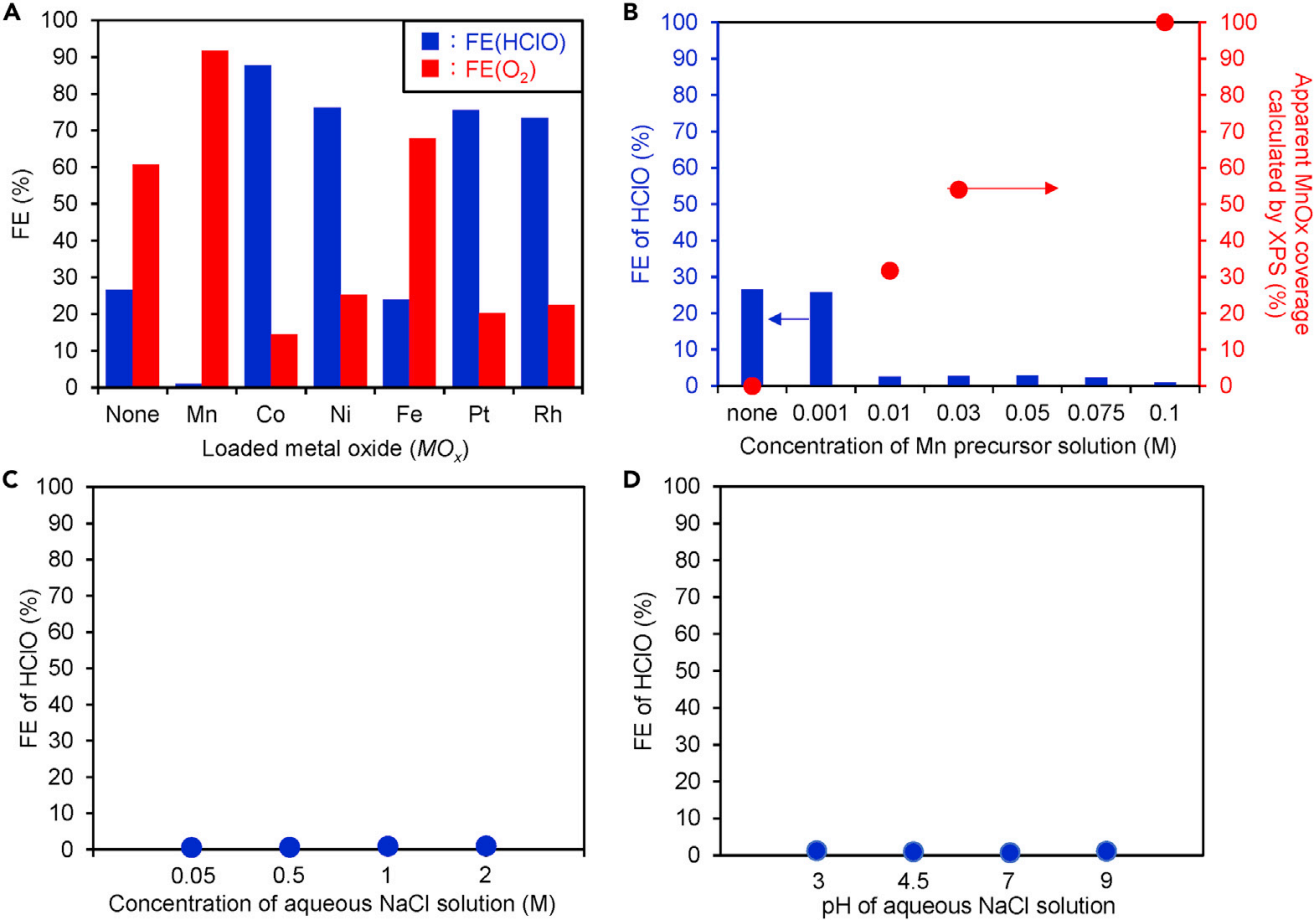
Photoelectrochemical Properties of *MO*_*x*_-Loaded Photoanodes (A) FEs for O_2_ and HClO generation on photoanodes (*MO*x/BiVO_4_/WO_3_/FTO) modified with and without various metal oxides. The concentrations of loaded metal precursor solution were 0.1 M for Mn, Co, Ni, and Fe and 0.03 M for Rh and Pt. The calcination temperature for loading was 400°C, at an electric charge of 2 C (1,000 s and a steady photocurrent of 2 mA) in a 0.5 M NaCl aqueous electrolyte (35 mL) under simulated solar light (AM-1.5, 1 SUN). (B) FE (HClO) and apparent MnO_*x*_-coverage calculated from XPS results for bare BiVO_4_/WO_3_/FTO and MnO_*x*_/BiVO_4_/WO_3_/FTO photoanodes, which were prepared by coating the manganese precursor solution with different concentrations (0.001–0.1 M) to change the MnO_x_ loading amount. (C) FE (HClO) on MnO_*x*_ (0.1 M)/BiVO_4_/WO_3_/FTO in aqueous NaCl solution with various concentrations (0.05-2 M). (D) FE (HClO) on MnO_*x*_ (0.1 M)/BiVO_4_/WO_3_/FTO anode in 0.5 M aqueous NaCl solution with various pH (pH 3–9).

A multilayer photoanode of BiVO_4_/WO_3_/FTO (FTO = F-doped SnO_2_ conducting glass), which has excellent performance for water splitting into H_2_ and O_2_ under low applied bias (Hong et al., 2011; Saito et al., 2012; Fuku et al., 2017), was used in this work. Figure 2A shows the faradaic efficiencies (FE) for O_2_ and HClO generation (FE(O_2_) and FE(HClO)) from aqueous NaCl solution on a BiVO_4_/WO_3_/FTO photoanode loaded with/without *MO*_*x*_ under simulated solar light (AM 1.5, 1 SUN). The potential required for the photo-electrolysis for steady current (2 mA) using these photoelectrodes was listed in Table S1. The bare photoanode produced both HClO and O_2_, and other oxidative by-products (Cl_2_, HClO_2_, HClO_3_ etc.) were not generated, as we recently reported (Iguchi et al., 2018). For the all photoanodes, the sum of FE for the oxidative products (O_2_ and HClO) became around 100% considering the measurement error (ca. 15%), which suggested the main products were O_2_ and HClO. O_2_ production with a high FE(O_2_) of >90% was observed on the MnO_x_/BiVO_4_/WO_3_/FTO photoanode, and no significant amount of HClO was detected with MnO_x_ loading. FE(HClO) with a CoO_x_-loaded photoanode was increased to ca. 88%. The dependence of FE(HClO) on the concentration of the Mn precursor solution and the concentration or pH of aqueous NaCl electrolyte solution were also investigated (Figures 2B–2D), and the CPR was effectively suppressed when the Mn precursor concentration was ≧0.01 M, the NaCl concentration was between 0.05 M–2 M, or the pH was between 3 and 9, respectively. Figure S1A shows current-potential (*I*-*V*) curves for the MnO_x_/BiVO_4_/WO_3_/FTO photoanodes under simulated solar light though a light chopper. The photocurrent could be observed on the photoanodes with or without MnO_x_ at much less than +0.88 V versus SHE, pH = 7. The optimum preparation temperature for MnO_*x*_ loading was 400°C for the best photocurrent among all MnO_x_-loaded photoanodes (Figure S1B). The photocurrent could be kept at almost 80% after MnO_x_ loading compared with that of the bare photoanode, because the color of the photoanode became brown and the photoabsorption might be inhibited by MnO_x_ loading (Figure S1C). Another possibility of decreased photocurrent could be considered to be producing some doped layers to accelerate charge recombination at the interface of MnO_x_/BiVO_4_ during the calcination process. We also examined the photoelectrochemical CPR in artificial seawater rather than in pure NaCl solution (Figure S2). The behavior of HClO selectivity in artificial seawater was very similar to that in pure NaCl solution (Figure 2A), and only the MnO_x_-loaded photoanode suppressed the CPR efficiently. The results suggest no significant effect of various anions and cations contained in the artificial seawater, such as SO_4_^2−^ and Mg^2+^, on the selectivity due to the low concentrations of these ions compared with Na^+^ and Cl^−^ (Desilvestro and Gräetzel, 1987; Luo et al., 2011; Barczuk et al., 2013; Fukuzumi et al., 2017; Iguchi et al., 2018). It was also confirmed that H_2_ was produced on a Pt cathode in artificial seawater with a high FE(H_2_) (ca. 100%).

### Characterizations of *MO*_*x*_/BiVO_4_/WO_3_/FTO

**Figure 3:**
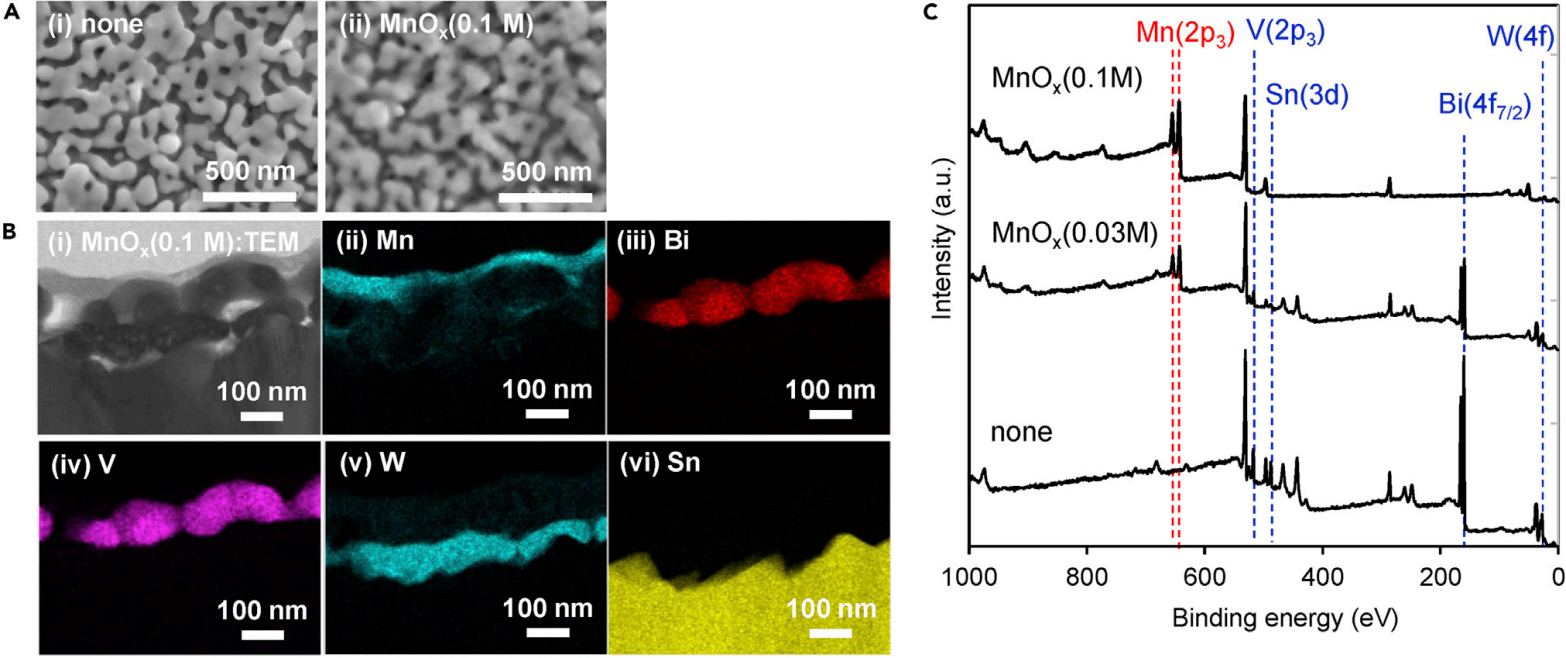
Characterization of BiVO_4_/WO_3_/FTO Photoanodes with and without Loading MnO_*x*_ (A–C) (A) Surface SEM images, (B) cross-sectional TEM-EDX images, and (C) XPS spectra of BiVO_4_/WO_3_/FTO photoanodes with and without MnO_*x*_. (A (ii) and B) The concentration of the MnO_*x*_ precursor solution was 0.1 M. (B) (i) TEM image and (ii-vi) EDX mapping images for each element.

The amounts of *MO*_*x*_ loading on the BiVO_4_/WO_3_/FTO photoanodes calculated from X-ray fluorescence (XRF) measurements are summarized in Table S2. The amount of MnO_x_ was 0.02 μmol/cm^2^, and those of other *MO*_*x*_ were in a similar range between 0.01 and 0.024 μmol/cm^2^. Surface scanning electron microscopic (SEM) observations of bare BiVO_4_/WO_3_/FTO (Figure 3A-(i)) confirmed a nanostructure with particle networks that consist of primary particles with diameters of ca. 100 nm. In contrast, the edge of the particles on the photoelectrode was not clear for MnO_x_(0.1 M)/BiVO_4_/WO_3_/FTO (Figure 3A-(ii)), which suggests the loaded MnO_x_ covered the entire surface of the photoanode. Transmission electron microscopic-energy dispersive X-ray spectroscopic (TEM-EDX) cross-sectional images of the photoanode loaded with MnO_x_ (0.1 M, Figure 3B) showed that a thin layer (ca. 10–30 nm thick) of MnO_x_ was present over the surface of the BiVO_4_/WO_3_/FTO photoanode. No electron diffraction pattern was obtained at the MnO_x_ part, which indicates that MnO_x_ was not crystalline. The apparent surface coverage of *MO*_*x*_ layers calculated from the peak area of X-ray photoelectron spectroscopy (XPS; Figure 3C) measurements are summarized in Table S3. In the case of the MnO_x_ (0.1 M)-coated sample, the peaks attributed to Bi, V, W, and Sn of the photoanode were not observed, whereas the peak attributed to Mn 2p_3_ was clear, which suggested the surface coverage of MnO_x_ was almost 100%. In contrast, for the MnO_x_ (0.03 M)-coated sample, the peaks attributed to Mn 2p_3_ decreased and peaks attributed to V 2p_3_, Bi 4f_7/2_, W 4f, and Sn 3d were observed, which indicates that MnO_x_(0.03 M) was not completely covering the photoanode surface, unlike that prepared with 0.1 M precursor solution. The dependence of the apparent surface coverage of MnOx on the concentration of manganese precursor solution is shown by the right-hand axis in Figure 2B. The coverage of MnO_x_ decreased on decreasing the concentration of the manganese precursor solution. Although the apparent surface coverage of MnO_x_ (0.01 M)-loaded BiVO_4_/WO_3_/FTO photoanode was only 30%, the selectivity toward the CPR was very low. Therefore, it is concluded that entire coverage of MnO_x_ on the BiVO_4_/WO_3_/FTO surface was not necessary to suppress the CPR. The results indicate that the selectivity toward HClO/O_2_ production could be controlled by this simple coating method using metal precursor solutions, and the CPR could be suppressed in aqueous solution containing Cl^−^ ions with a photoanode surface partially loaded with MnO_x_.

### Properties of *MO*_*x*_/FTO under Dark Conditions

The electrochemical properties of the simple structured-*MO*_*x*_/FTO anodes without semiconductor layers were also evaluated under dark conditions (Figure S3). We checked the product amount of HClO/O_2_ and calculated the values of FE(HClO), FE(O_2_) on bare FTO, and *MOx*/FTO, confirming that the sum of the FE(HClO) and FE(O_2_) became almost 100% within variation of measurement errors in each electrode. FE(HClO) for various *MO*_*x*_/FTO anodes (Figure S3A) had a similar tendency to that for the *MO*_*x*_/BiVO_4_/WO_3_/FTO photoanodes (Figure 2A). It was also confirmed that MnO_x_ loading of the anodes effectively suppressed the CPR in aqueous NaCl solution under a wide range of conditions, regardless of the Mn precursor solution concentration (0.01–0.1 M), the NaCl concentration (0.005–2 M), pH (3–9), applied bias (1.5–1.8 V versus SHE, pH = 7), the valence or structure of MnO_x_ (amorphous, Mn_2_O_3_ and Mn_3_O_4_), preparation methods of MnO_*x*_ such as calcination temperature (300–550°C), and the kinds of manganese salt dissolving the precursor solution, as shown in Figures S3B–S3I. SEM images, MnO_x_ coverages, and FE(HClO) of MnO_x_/FTO anodes are shown in Figure S4. In the case of the MnO_x_ (0.03 M)/FTO with very low FE(HClO), SEM observations indicated that MnO_x_ did not entirely coat the surface of FTO, and the apparent coverage of MnO_x_ (0.03 M) calculated from the XPS spectrum was only 65%. It is suggested that complete coating of MnO_x_ on FTO anodes was not needed to suppress the CPR, as well as photoanodes.

### Mechanism of Selective O_2_ Production on MnO_x_-Loaded Photoanode

There are two possibilities why the MnO_x_ loaded on the photoanodes or anodes could suppress the CPR; direct O_2_ production on MnO_x_ and indirect O_2_ production through the decomposition of HClO as an intermediate. The HClO initially added to the NaCl aqueous solution was not decomposed on a MnO_x_/FTO anode under dark conditions (Figure S5). This suggests that indirect O_2_ production on MnO_x_/FTO by HClO decomposition on the MnO_x_ surface can be denied and that O_2_ was directly produced on MnO_x_/FTO in NaCl aqueous solution.

**Figure 4:**
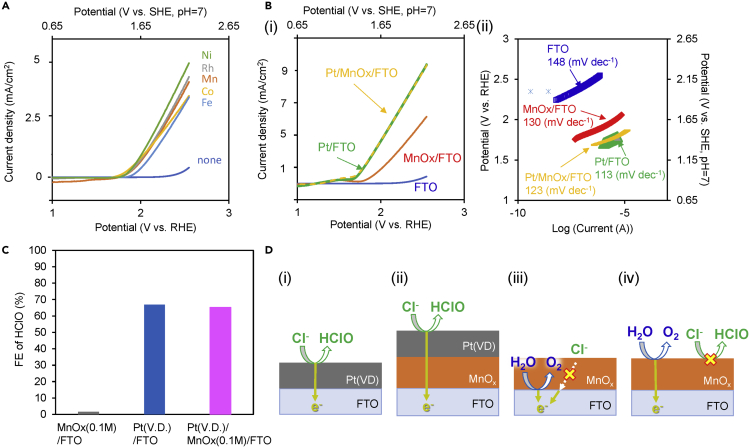
Evaluation of Electrochemical Properties and Schematic Diagrams on O_2_/HClO Production on *MO*_*x*_/FTO Anodes (A) *I*-*V* characteristics for bare FTO and various *MO*_x_ (0.1 M)*/*FTO anodes measured in 0.5 M NaCl aqueous solution (sweeping in the cathodic direction). (B) (i) *I*-*V* characteristics and (ii) Tafel plots for various anodes (FTO, MnO_*x*_ (0.1 M)/FTO, Pt(VD)/FTO, and Pt(VD)/MnO_*x*_ (0.1 M)/FTO) measured in 0.5 M NaCl aqueous solution. The Pt (VD) layer was prepared using the vapor deposition method. (C) FE (HClO) for various anodes at an electric charge of 2 C (1,000 s at a steady current of 2 mA) in a 0.5 M aqueous NaCl solution. (D) Schematic diagrams of (i) Pt (VD)/FTO and (ii) Pt (VD)/MnO_*x*_ (0.1 M)/FTO anodes. (iii) Cl^−^-impermeable mechanism and (iv) catalytic mechanism for MnO_*x*_ (0.1 M)/FTO anodes.

In the case of noble metal anodes such as IrO_2_, selective OER from an aqueous solution containing Cl^−^ ions can be realized by coating an MnO_x_ layer on the entire surface of the anode (Bennet, 1980; El-Moneim et al., 2009; Vos et al., 2018; Ibrahim and Canan, 2015; Gidon et al., 2018). In the field of water electrolysis, this selective OER has been explained by a “Cl^−^-impermeable mechanism,” where Cl^−^ ions cannot reach the surface of the noble metal through the MnO_x_ layer, rather than by a “catalytic mechanism” where MnO_x_ itself functions as an electrocatalyst for O_2_ production (Vos et al., 2018). In this Cl^−^-impermeable mechanism, the MnO_x_ layer must completely cover the entire surface of the noble metal, and no oxidation reaction should occur on the MnO_x_ but rather on the noble metal catalyst, which indicates that the reaction sites are completely different with each mechanism. However, in this study, much data that cannot be explained by the Cl^−^-impermeable mechanism was obtained. First, current-voltage (*I*-*V*) curves for the MnO_x_/FTO anode showed that the onset potential was significantly shifted to lower potential compared with that for bare FTO (Figures 4A and 4B). Second, complete coating of MnO_x_ on FTO anode was not necessary to suppress the CPR (Figures S3B and S4). Third, it was confirmed whether or not the oxidation reaction occurred on MnO_x_ (Figures 4C and 4D). A thick MnO_x_ (0.1 M) layer that covered the entire FTO surface was prepared, and then it was coated with Pt by vapor deposition method (Pt (VD)/MnO_x_/FTO). The *I*-*V* properties and Tafel slope were improved by Pt (VD) loading (Figure 4B-(ii)). The FE (HClO) on the Pt (VD)/MnO_x_/FTO anode (65.2%) was almost same as that on the Pt (VD)/FTO anode (66.9%) and was completely different from that on MnO_x_/FTO (<1.6%), as shown in Figure 4C, which indicates that the selectivity and *I*-*V* properties could be controlled by modification of the top surface. These results suggest that the oxidation reaction occurred on the top surface of MnO_x_ on the MnO_x_ (0.1 M)/FTO anode, and not on the surface of FTO (Figure 4D). Furthermore, the Cl^−^ adsorption characteristics of *MO*_*x*_ were compared using XPS, and Cl^−^ ions were found not to be adsorbed by CoO_x_, FeO_x_, NiO_x_, or MnO_x_ (Table S4, Cl coverage <1%). Therefore, it was difficult to explain MnO_x_ peculiarity by the difference of the adsorption characteristics of Cl^−^. From all these results, we can conclude that the mechanism for the selectivity toward the OER/CPR on MnO_x_/FTO from an aqueous solution containing Cl^−^ ions can be explained not by the Cl^−^-impermeable mechanism but rather by the catalytic mechanism (Figure 4D-(iii and iv)).

**Figure 5:**
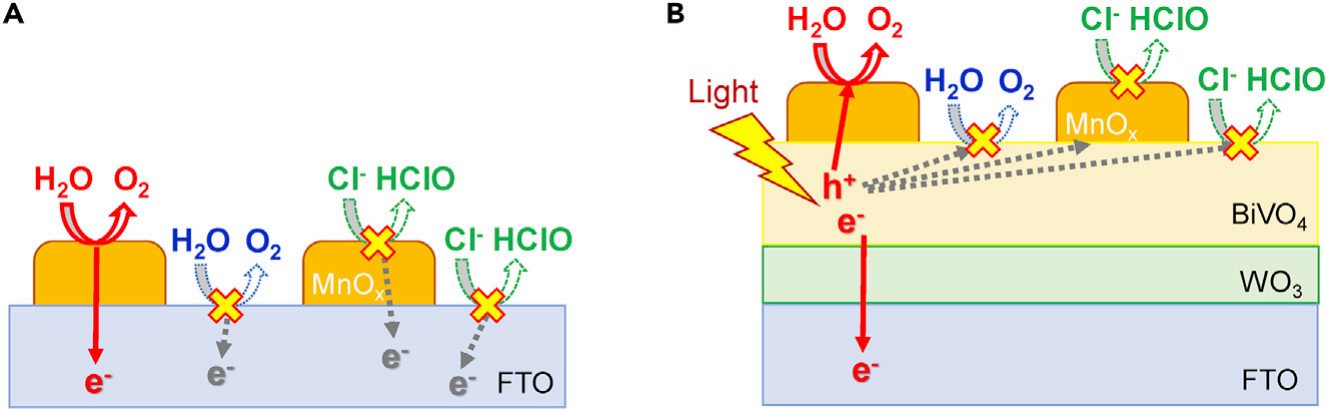
Schematic Diagrams on O_2_/HClO Production over Partially Loaded MnO_x_ Catalyst (A and B) The OER and CPR on (A) FTO anode and (B) BiVO_4_/WO_3_/FTO photoanode partially loaded with MnO_x_ catalyst. The selectivity is controlled by the relative relationship of the overpotentials for each reaction. The overpotential for the OER on MnO_x_ might be much lower than those of other reactions such as the CPR on MnO_x_, and the OER and CPR on BiVO_4_.

The special OER selectivity on the MnO_x_ catalyst-loaded anodes might be explained by the difference or the ratio of the overpotential with the OER and CPR reactions in each electrolyte solution. The potentials required for steady current electrolysis on various *MO_x_*/FTO were listed in Table S5. The behaviors of potential on each anode were similar in NaCl and NaH_2_PO_4_ aqueous solutions except for unstable anodes; the precious metals showed the lowest potential level, and CoO_x_, FeO_x_, NiO_x_, and MnO_x_ showed the second lowest level. On the other hand, the evaluation of each specific overpotential in NaCl aqueous solution is difficult because both the OER and CPR occur. It should be emphasized again that the FE (HClO) was very low even when MnO_x_ was not fully coated on FTO; therefore, it is surmised that the overpotential for the OER on MnO_x_ was much lower than those of the other reactions such as the CPR on MnO_x_, and the OER and CPR on FTO relatively in NaCl solution (Figure 5A). As for the photoanode reactions using *MO*_*x*_/BiVO_4_/WO_3_/FTO, the specificity of MnO_x_ could not be explained by the decomposition of HClO produced into O_2_ by UV light excitation from solar light as reported (Fukuzumi et al., 2017), because the results in Figure 2A were compared under the same irradiation conditions. It was confirmed that the HClO selectivity was very low, even when the apparent coverage of MnO_x_ was only 30%, as shown in Figure 2B. Considering the analogy with the results for the MnO_x_/FTO anode, the Cl^−^-impermeable mechanism for MnO_x_ loaded on a photoanode can also be excluded. The turnover number (ca. 154) of e^−^ to Mn on the MnO_x_-loaded photoanode was much more than 1. Therefore, it is reasonable to explain the results by the catalytic mechanism, where photoexcited h^+^ on BiVO_4_ immediately moves to MnO_x_ and the preferential oxidation of water on the MnO_x_ surface into O_2_ occurs without the CPR (Figure 5B). It should be noted that various MnO_x_ catalysts such as amorphous Mn_2_O_3_, Mn_3_O_4_, and Ca-MnO_x_ could suppress the CPR (Figure S3G), which indicates that manganese oxides themselves may have specificity with a large overpotential ratio for the CPR to the OER in NaCl solution. This catalytic mechanism of MnO_x_ is generally that for the OER on Mn_4_O_5_ core cluster with Ca in the photosystem II (PSII) of natural photosynthesis (Suga et al., 2017; Zhang et al., 2018; Zaharueva et al., 2011; Najafpour et al., 2010); therefore, it can also be a common mechanism in the fields of electrolysis and artificial photosynthesis using photoanodes. Currently, the intrinsic reason for the specific dependence of the overpotentials on these elements including manganese is not clear, and the comparison of energy barriers of transition state between CPR and OER on a certain model structure of each *MO*_*x*_ through computational chemistry is under investigation.

### Evolutional Hypothesis for the Mn_4_O_5_ Core of Natural Photosynthesis

Natural OER metal centers of cyanobacteria, algae, or plants containing elements other than Mn have not been found to date. Mn has many advantages such as a low overpotential for OER and earth-abundant transition metal element with various valence states (Zhang et al., 2018; Armstrong., 2008; Sauer and Yachandra, 2002; Raveau and Seikh, 2012; Bryan et al., 2016). On the other hand, some metal oxides such as Fe, Ni, and Co also basically satisfy all these requirements (Zaharueva et al., 2011; Raveau and Shiek, 2012; Bryan et al., 2016). Therefore, there is no hypothesis to explain why Fe, Ni, or Co is not used as the OER center in PSII so far. Based on our specific results that MnO_x_ catalyzes the OER selectively from NaCl aqueous solution under a wide range of conditions, even at low pH and low Cl^−^ concentration compared with those for natural photosynthesis *in vivo* (ca. 100 mM of Cl^−^ in chloroplasts) (Robinson and Downton, 1984; Baranov and Haddy, 2017; Jagendorf and Uribe, 1966; Beebo et al., 2013), it is possible to advocate a new hypothesis as to why MnO_x_ is selected as the OER center of PSII that appeared on the ancient earth. It is because HClO is significantly toxic to organisms, and Mn is the only element that can produce only O_2_ without the generation of HClO in aqueous solution containing Cl^−^ ions under various conditions *in vivo*. Considering that HClO has extremely strong bactericidal properties compared with H_2_O_2_ (McKenna and Davies, 1988), and that it should be necessary to avoid the generation of even a small amount of HClO in *vivo*, the specialty of natural Mn_4_O_5_ can be comprehensively explained by the results of this study.

### Limitation of the Study

We investigated the O_2_/HClO production in aqueous solution containing Cl^−^ on BiVO_4_/WO_3_/FTO photoanodes and FTO anodes modified with various *MO_x_*. It is emphasized that the modification of partially loaded MnO_x_ resulted in specifically catalyzing the OER without HClO production over a wide range of conditions. These results will not only provide some insight into the practical application of artificial photosynthesis technologies using salted water and solar energy but also facilitate the elucidation of the function of Mn_4_O_5_ core cluster with Ca in natural photosynthesis. Considering our results, one hypothesis about the evolutional process of Mn_4_O_5_ core cluster in natural PSII can be proposed; HClO is toxic to bacteria, so that manganese element might be selected by nature due to the unique catalysis on MnO_x_, where HClO production is fully suppressed. Complete elucidation of evolutional hypothesis is difficult generally; however, our hypothesis will be elucidated by various ways. For example, we have some plans about the investigation on the effect of coordination of organic compounds with carboxyl and amino groups, mimicking the atmosphere in the protein of PSII, on various nano-size *MO_x_* and multinuclear metal complexes as electrocatalysts systematically, as well as investigation through computational chemistry in the future.

### Resource Availability

#### Lead Contact

Further information and requests for resources should be directed to the Lead Contact, Kazuhiro Sayama (k.sayama@aist.go.jp).

#### Materials Availability

This study did not generate new unique reagents.

#### Data and Code Availability

This study did not generate/analyze datasets/code.

## Methods

All methods can be found in the accompanying Transparent Methods supplemental file.

## Supplemental Information

Document S1. Transparent Methods, Figures S1–S5, and Tables S1–S5
