# Supplementary material for: Functions of MnOx in NaCl Aqueous Solution for Artificial Photosynthesis
Source: iScience. 2020 Oct 8;23(10):101540. doi: 10.1016/j.isci.2020.101540 (PMC7584672; doi:10.1016/j.isci.2020.101540)
Supplement: Document S1. Transparent Methods, Figures S1–S5, and Tables S1–S5 [file mmc1.pdf]

iScience, Volume *23*

Supplemental Information

Functions of MnO_x in NaCl Aqueous Solution for Artificial Photosynthesis

Sayuri Okunaka, Yugo Miseki, and Kazuhiro Sayama

Supplemental Information

Table of Contents

This PDF file includes:
Supplementary Figures and Captions

Transparent Methods
Table S1 Potential required for photo-electrolysis for steady current (2 mA) using various *MO*$_x$/$BiVO_4$/WO_3/FTO photoelectrodes.
Table S2 Amount of *MO*$_x$ loading on the $BiVO_4$/WO_3/FTO photoanodes
Table S3 Apparent *MO*$_x$-coverage (%) for the $BiVO_4$/WO_3/FTO photoanodes
Table S4 Amounts of adsorbed Cl^- ions on the *MO*$_x$/FTO after dipped in NaCl aq.
Table S5 Potential required for electrolysis for steady current (2 mA) using various *MO*$_x$/FTO electrodes.
Figure S1 Photoelectrochemical performance and optical property of photoanode.
Figure S2 FE(HClO) on photoanodes in 0.5 M of NaCl aq. and artificial seawater.
Figure S3 FEs for HClO generation on *MO*$_x$/FTO under dark conditions.
Figure S4 SEM images and MnO_x coverages of bare and MnO_x/FTO anodes.
Figure S5 HClO degradation behavior for MnO_x (0.1 M)/FTO under dark conditions.
References

Transparent Methods

Materials

Metal organic solutions of Mn, Co, Fe, Ni were purchased from Symetrix Co., USA (purchased from Koujundo Chemical. Co., Japan). $H_2PtCl_6 \cdot 6H_2O$ and $Rh(NO_3)_3$, $IrCl_3$, $AgNO_3$, NaCl, NaH_2PO_4 were purchased from Wako Pure Chemical, Japan. $Pd(NO_3)_2$, RuCl were purchased from Nakalai Tesque. All reagents were used as received, and all the experiments were carried out under ambient condition without eliminating the moisture from the atmosphere.

Preparation of $BiVO_4/WO_3$/FTO photoanode

$BiVO_4/WO_3$/FTO photoanodes were prepared as follows. Precursor solutions of each oxide semiconductor were coated on F-doped SnO_2 conductive glass substrate (FTO; 10 Ω sq^{-1}, Nippon Sheet Glass Co.) by spin-coating and then calcination for each coating. A WO_3 underlayer was coated on the FTO substrate by spin-coating (1500 rpm, 15 s) aqueous peroxo-tungstic acid solution (1.4 M) containing W ions, followed by calcination at 500 °C for 30 min in air. The $BiVO_4$ layer on WO_3/FTO was also fabricated by spin-coating (1000 rpm, 15 s) using a Bi and V precursor solution, followed by calcination at 550 °C for 30 min in air. The $BiVO_4$ precursor solutions was a mixed solution of 0.2 M Bi^{3+} and 0.2 M V^{5+} in a 1:1 volume ratio diluted with butyl acetate.

Preparation of $MO_x/BiVO_4/WO_3$/FTO photoanodes and MO_x/FTO anodes

The MO_x (where M = Mn, Co, Fe, Ni, Pt, Rh) layers on the $BiVO_4/WO_3$/FTO photoanodes (denoted as $MO_x/BiVO_4/WO_3$/FTO) or FTO anodes (denoted as MO_x/FTO) were prepared by spin-coating (1500 rpm, 15 s) metal organic solutions in butyl acetate on the $BiVO_4/WO_3$/FTO and calcination at 400 °C in air for 1 h. In the case of loading the non-noble metals, metal organic solutions (Symetrix Co., USA, purchased from Koujundo Chemical. Co., Japan) in butyl acetate (0.1 M) were used. In the cases of loading with noble metals, aqueous solutions of $H_2PtCl_6 \cdot 6H_2O$ (Wako), $Rh(NO_3)_3$ (Wako) were prepared. It is difficult to prepare high concentration solutions of these noble metal precursor salts; therefore, the concentration of these solutions was adjusted to 0.03 M.

The expression MO_x was used, regardless the valence of the metal. To evaluate the reaction sites, Pt (VD) was coated onto FTO or MnO_x (0.1 M)/FTO by vapor deposition method under vacuum (JFC-1600, JEOL, emission current: 40 mA, emission time: 7 min, sample distance: 30 mm).

For the electrodes used in the electrolysis system under dark conditions, FTO anode electrodes modified with various metal oxides (MO_x/FTO, where M = Mn, Co, Fe, Ni, Cu, Ag, Pt, Pd, Rh, Ru) were prepared. In the case of loading the non-noble metals, metal organic solutions in butyl acetate (0.03 or 0.1 M) were used. In the cases of loading with noble metals, aqueous solutions of $H_2PtCl_6 \cdot 6H_2O$, $Rh(NO_3)_3$, $Pd(NO_3)_2$, RuCl, $AgNO_3$ and $IrCl_3$ were prepared. It is difficult to prepare high concentration solutions of these noble metal precursor salts; therefore, the concentration of these solutions was adjusted to 0.03 M. The MO_x layers on the FTO electrodes (denoted as MO_x/FTO) were prepared by spin-coating (1500 rpm, 15 s) metal organic solutions in butyl acetate on the FTO and calcination at 400 °C in air for 1 h.

To examine the effect of manganese species on FE (HClO), Mn_2O_3/FTO, Mn_3O_4/FTO,

and Ca-MnO_x/FTO anodes were prepared as follows. A Mn_2O_3/FTO anode was prepared by electrochemical deposition (ED-S) in a solution of 0.25 M $MnSO_4$ and 0.25 M Na_2SO_4 (1:1 v/v) for 10 min. The films were rinsed with water and dried, followed by calcination at 400 °C for 1 h in air (Ramírez. et al. 2014). The Mn_3O_4/FTO anode was prepared by electrochemical deposition (ED-N) in a solution of 0.25 M $Mn(NO_3)_2$ and 0.25 M Na_2SO_4 (1:1 v/v) for 10 min at 70 °C. (Qi. et al. 2016) The films were rinsed with water and dried at 80 °C for 1 h in air. A Ca-MnO_x(Mn/Ca=4) anode was prepared by spin-coating (1500 rpm, 15 s) metal organic solutions (0.1 M, Ca:Mn = 1:4 molar ratio, Symetrix Co., USA, purchased from Koujundo Chemical. Co., Japan) in butyl acetate on the FTO and calcination at 400 °C in air for 1 h.

Characterization

The anode samples obtained were characterized using X-ray diffraction (XRD; PANalytical, EMPYREAN, rotating anode diffractometer, 40 kV, 10 mA) with Cu $K\alpha$ radiation ($\lambda_{K\alpha}$ = 1.5406Å), X-ray fluorescence (XRF; Rigaku, Supermini200), X-ray photoelectron spectroscopy (XPS; Ulvac Co., XPS-1800), scanning electron microscopy (SEM; HITACHI, S-4100), and transmission electron microscopy (TEM; Hitachi High-Technologies Co., Ltd., HD2700) with energy dispersive X-ray spectroscopy (EDX) mapping. The MnO_x coverage was calculated using the spectrum area obtained from XPS spectrum and intensity factor of Mn to the sum of those of Mn, Bi, V, W and Sn.

Photoelectrochemical and electrochemical production of HClO and O_2

Photoelectrochemical properties

The photoelectrochemical performance of the photoanodes was measured using an electrochemical analyzer (BAS. Inc., ALS 760E) and a solar simulator (SAN-EI ELECTRIC Co., XES-151S) calibrated to AM-1.5 (1 SUN, 100 mW cm^{-2}) with a spectroradiometer (SOMA Optics, Ltd., Model S-2440). The irradiation area (0.28 cm^2) was defined using a black mask. The simulated solar light was irradiated from the semiconductor side. The current–voltage (*I–V*) characteristics were measured using a two-compartment cell (Pyrex-made) with an ion-exchange membrane (SELEMION, AGC Engineering), and a photoanode equipped with a back-reflection plate as the working electrode, an Ag/AgCl electrode as the reference electrode, and a Pt wire as the counter electrode The scan rate was 50 mV s^{-1}. The measured potentials vs. Ag/AgCl were converted to the standard hydrogen electrode (SHE) and the reversible hydrogen electrode (RHE) scale according to:

$$E_{SHE} = E_{Ag/AgCl} + 0.206 \qquad \text{(S1)}$$

$$E_{RHE} = E_{Ag/AgCl} + 0.059pH + E^{o}{}_{Ag/AgCl} \qquad \text{(S2)}$$

where E_{SHE} and E_{RHE} are the converted potential vs. SHE or RHE, $E^{o}_{Ag/AgCl}$ = 0.1976 at 25 °C, and $E_{Ag/AgCl}$ is the experimentally measured potential against the Ag/AgCl reference. An aqueous solution of 0.5 M NaCl (35 mL, pH = 5.9) was used as the electrolyte.

Electrochemical properties

The current–voltage (I–V) characteristics on the FTO and MO_x/FTO anodes were measured using a two-compartment cell (Pyrex-made) with an ion-exchange membrane (SELEMION, AGC Engineering), and an anode as the working electrode, an Ag/AgCl electrode as the reference electrode, and a Pt wire as the counter electrode. The scan rate was 50 mV s^{-1}. The measured potentials vs. Ag/AgCl were converted to the SHE and the RHE. An aqueous solution of 0.5 M NaCl (35 mL, pH = 5.9) was used as the electrolyte.

Simultaneous production of HClO and O_2

The simultaneous production of HClO and O_2 on the photoanodes was measured using an electrochemical analyzer (BAS. Inc., ALS 760E) and a solar simulator (SAN-EI ELECTRIC Co., XES-151S) calibrated to AM-1.5 (1 SUN, 100 mW cm^{-2}) with a spectroradiometer (SOMA Optics, Ltd., Model S-2440). The simulated solar light was irradiated from the semiconductor side. The reaction on the obtained photoanodes was performed under solar light irradiation using a two-compartment cell, equipped with an ion-exchange membrane (SELEMION, AGC Engineering) between the anode and cathode. Pt wire was used as a counter electrode to effectively reduce water to H_2. An aqueous solution (anode: 35 mL; cathode: 35 mL) of NaCl (0.5 M) was used as the electrolyte. In the photoelectrochemical reaction, photoanodes (1.5×5 cm) were used. Each solution was stirred by magnetic stirring at ca. 400 rpm. In the case of the FTO and MO_x/FTO anodes, the production of HClO in the dark was conducted by the same method.

The concentration of HClO produced was measured with a UV-vis spectrometer (JASCO, Y-730) using the *N,N*-diethyl-*p*-phenylenediamine (DPD) method. The faradaic efficiencies (FEs) of HClO (FE (HClO)) can be calculated as:

$$FE(HClO) = \frac{(amount\ of\ generated\ HClO)}{(theoretical\ amount\ of\ HClO)} \times 100 = \frac{(amount\ of\ generated\ HClO)}{(amount\ of\ generated\ electrons/2)} \times 100 \quad \text{(S3)}$$

The amount of O_2 evolved in both the liquid and gas phases were determined using an O_2 sensor (Optical Oxygen Meter, Pyro Science and FireStingO_2) and combining each value. These HClO/O_2 production reactions of the main part were measured 3 times, and the reproducibility of the data was confirmed with the variation of experimental errors within 10%. The amount of hydrogen evolved from Pt electrode was determined with a gas chromatograph equipped with a TCD detector. The faradaic efficiency of H_2 (FE (H_2)) can be calculated as:

$$FE(HClO) = \frac{(amount\ of\ generated\ H_2)}{(theoretical\ amount\ of\ H_2)} \times 100 = \frac{(amount\ of\ generated\ H_2)}{(amount\ of\ generated\ electrons/2)} \times 100 \quad \text{(S4)}$$

Results

Table S1. Potential required for photo-electrolysis for steady current (2 mA) using various *MO_x*/$BiVO_4$/WO_3/FTO photoelectrodes. Related to Figure 2.

***MO_x*/$BiVO_4$/WO_3/FTO**	**Potential (V vs. SHE, pH=7)**
None	0.74
Mn	0.83
Co	0.68
Ni	0.70
Fe	0.78
Pt	0.65
Rh	0.80

Potential required for electrolysis with an electric charge of 2 C in 0.5 M aqueous NaCl solution using various *MO_x*/ $BiVO_4$/WO_3/FTO electrodes (1.5 cm x 5 cm). The concentration of loaded metal precursor solution was 0.03 M.

Table S2. Amount of *MO_x* loading on the $BiVO_4$/WO_3/FTO photoanodes. Related to Figure 2.

MO_x	***MO_x* loading (μmol/cm^2)**
None	0
Mn	0.020
Co	0.014
Fe	0.017
Ni	0.024
Rh	0.016
Pt	0.010

Amount of *MO_x* loading on the $BiVO_4$/WO_3/FTO photoanodes was calculated using XRF spectra. The concentrations of loaded metal precursor solutions were (a) 0.1 M for Mn, Co, Ni, and Fe, and (b) 0.03 M for Rh and Pt.

Table S3. Apparent *MO_x*-coverage (%) for the $BiVO_4$/WO_3/FTO photoanodes. Related to Figure 2.

MO_x	**Coverage (%)**	
	**(A) 0.03 M**	**(B) 0.1 M**
Mn	54.0	100.0
Co	51.6	79.9
Ni	77.6	91.9
Fe	79.3	87.4
Rh	32.1	-
Pt	38.5	-

Apparent *MO_x*-coverage (%) calculated from XPS spectra for $BiVO_4$/WO_3/FTO photoanodes modified with various metal oxides (*MO_x*). The concentrations of the coated metal precursor solutions were (A) 0.03 M for Mn, Co, Ni, Fe, Rh and Pt, and (B) 0.1 M for Mn, Co, Ni, and Fe. The MnO_x coverage was calculated using the spectrum area and intensity factor of Mn to

the sum of those of Mn, Bi, V, W and Sn.

Table S4. Amounts of adsorbed Cl^- ions on the MO_x/FTO after dipped in NaCl aq. Related to Figure 4.

***MO*$_x$**	**Amount of Cl (%) after immersion in NaCl aq.**
None	0.2
Mn	0.2
Co	< 0.1
Ni	0.5
Fe	0.6
Pt	3.2
Rh	3.5

Amounts of adsorbed Cl^- ions on the MO_x/FTO anode after immersion in 10 mL of 0.5 M aqueous NaCl solution for 3 h, determined from XPS measurements. Although the amount of Cl^- adsorption on Pt and RhO_x on the FTO was slightly larger than that of other typical metals, this is because the precursor solutions of Pt and Rh contain Cl^- ions.

Table S5. Potential required for electrolysis for steady current (2 mA) using various MO_x/FTO electrodes. Related to Figure 4.

***MO*$_x$/FTO**	**(A) Potential (V vs. SHE) in NaCl aq.**	**(B) Potential (V vs. SHE) in NaH_2PO_4 aq.**
None	2.43	2.45
Mn	1.62	1.40
Co	1.50	1.38
Ni	1.61	1.50*
Fe	1.67	1.44
Cu	1.95*	2.23*
Cr	1.95*	2.19*
Ag	1.74	1.86
Pt	1.23	1.34
Pd	2.04*	1.50
Rh	1.38	1.37
Ru	1.35	1.36
Ir	1.30	1.36

Potential after the electrolysis with an electric charge of 2 C (1000 s, in a steady current of 2 mA) in (A) 0.5 M aqueous NaCl solution (pH 5.9) and (B) 0.5 M aqueous NaH_2PO_4 solution (pH 4.3) using various MO_x/FTO electrodes (1.5 cm x 4 cm). The concentration of loaded metal precursor solution was 0.03 M. *Elution was observed during the reaction.

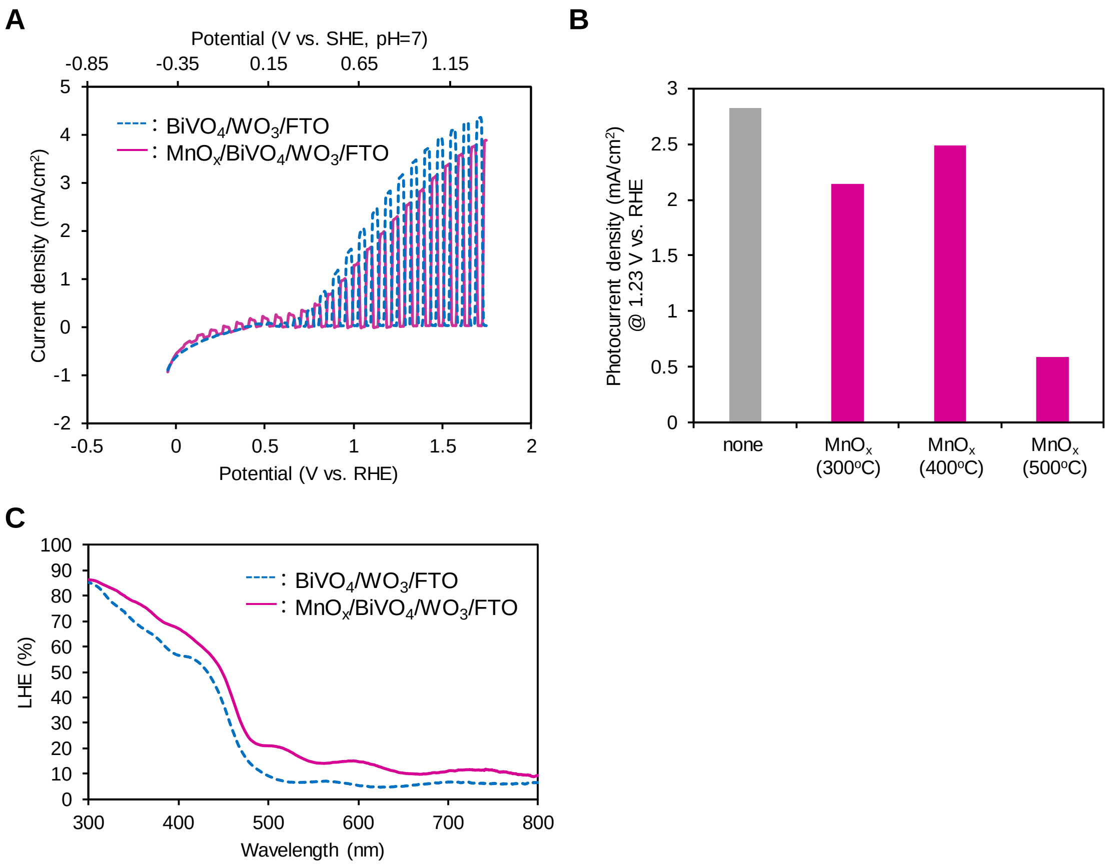

Figure S1. Photoelectrochemical performance and optical property of photoanode. Related to Figure 2.

(A) *I-V* characteristics for bare $BiVO_4/WO_3$/FTO and MnO_x(0.1M)/$BiVO_4/WO_3$/FTO photoanodes measured in 0.5 M NaCl aqueous solution under simulated solar light (AM-1.5, 1 SUN, irradiation area: 0.28 cm^2, irradiated from the semiconductor side) with a light chopper. MnO_x was prepared using 0.1 M-Mn precursor solution and by calcination at 400 °C. (B) Photocurrent at + 0.88 V vs. SHE at pH=7 of the bare $BiVO_4/WO_3$/FTO and MnO_x(0.1M)/$BiVO_4/WO_3$/FTO photoanodes, which were calcined at different temperatures (300 – 500 °C) after coating of the manganese precursor solution, measured in 0.5 M NaCl aqueous solution under simulated solar light. (C) Light harvesting efficiency (LHE) spectra for the bare $BiVO_4/WO_3$/FTO and MnO_x(0.1M)/$BiVO_4/WO_3$/FTO photoanodes calcined at 400 °C after coating with the manganese precursor solution. LHE was calculated from transmittance (T, %) and reflectance (R, %) using the following formula:

$$LHE(\%) = 100 - T - R \qquad \text{(S5)}$$

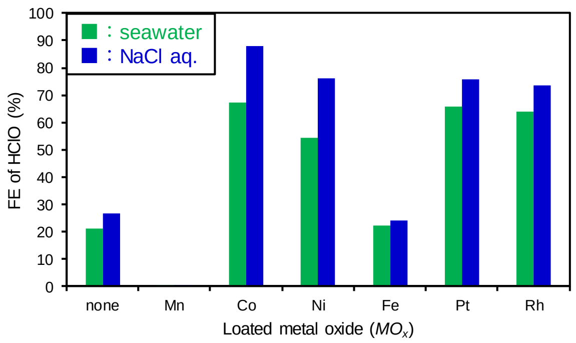

Figure S2. FE(HClO) on photoanodes in 0.5 M of NaCl aq. and artificial seawater. Related to Figure 2.

FEs for oxidative HClO generation on photoanodes ($MO_x/BiVO_4/WO_3$/FTO) modified with and without various metal oxides at an electric charge of 2 C (1000 s at steady photocurrent of 2 mA) in 0.5 M of NaCl aq. and artificial seawater (35 mL) under simulated solar light (AM-1.5, 1 SUN). The concentrations of coated metal precursor solutions were 0.1 M for Mn, Co, Ni, and Fe, and 0.03 M for Rh and Pt. The artificial seawater (MARINE ART SF-1) was purchased from Osaka Yakken. Co. Ltd., Japan (Iguchi. et al. 2018).

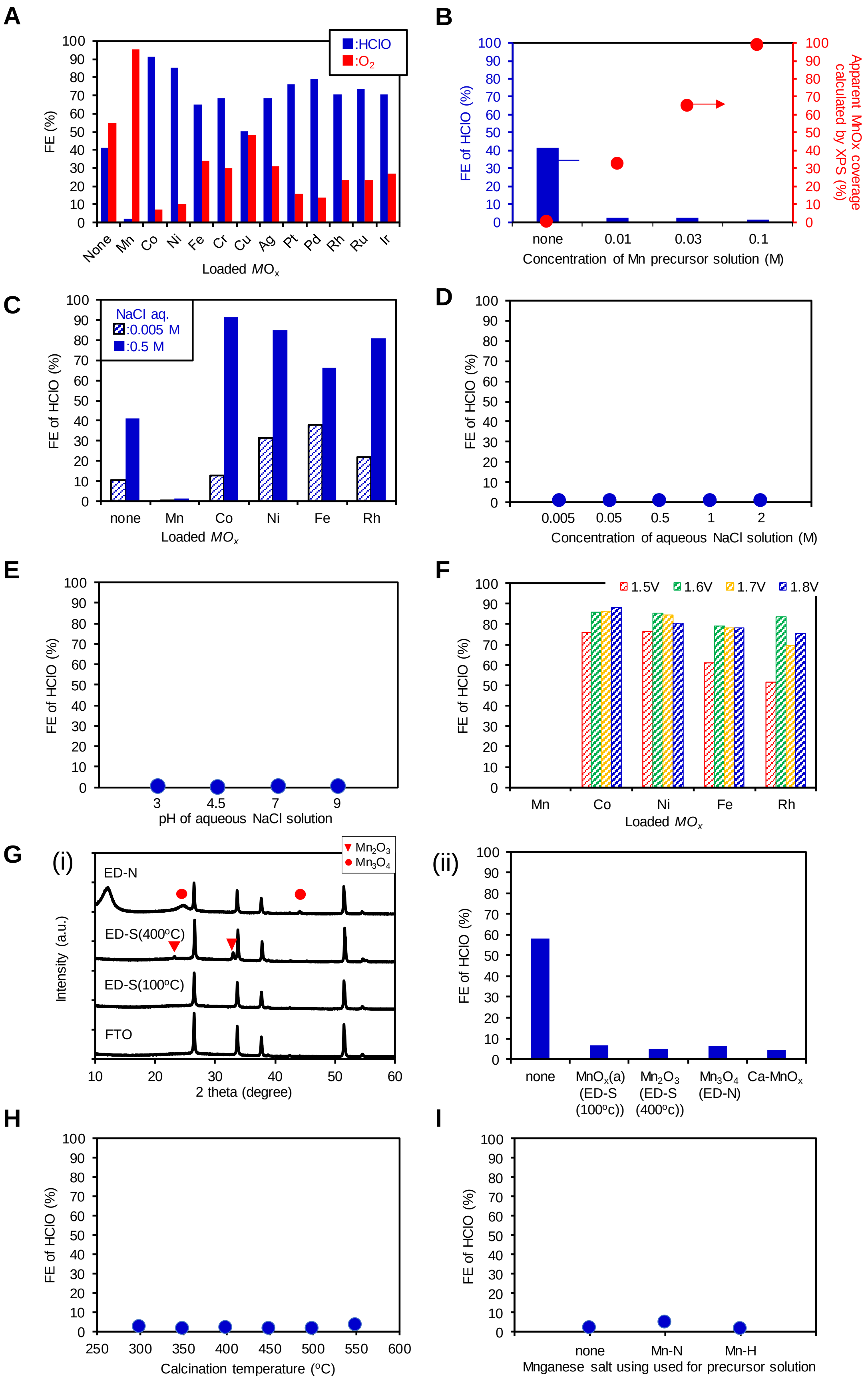
A
FE (%)
:HClO
:O2
None
Mn
Co
Ni
Fe
Cr
Cu
Ag
Pt
Pd
Rh
Ru
Ir
Loaded MOx
B
FE of HClO (%)
Apparent MnOx coverage calculated by XPS (%)
none
0.01
0.03
0.1
Concentration of Mn precursor solution (M)
C
FE of HClO (%)
NaCl aq.
:0.005 M
:0.5 M
none
Mn
Co
Ni
Fe
Rh
Loaded MOx
D
FE of HClO (%)
0.005
0.05
0.5
1
2
Concentration of aqueous NaCl solution (M)
E
FE of HClO (%)
3
4.5
7
9
pH of aqueous NaCl solution
F
FE of HClO (%)
1.5V
1.6V
1.7V
1.8V
Mn
Co
Ni
Fe
Rh
Loaded MOx
G
(i)
Mn2O3
Mn3O4
ED-N
ED-S(400°C)
ED-S(100°C)
FTO
Intensity (a.u.)
2 theta (degree)
(ii)
FE of HClO (%)
none
MnOx(a) (ED-S (100°c))
Mn2O3 (ED-S (400°c))
Mn3O4 (ED-N)
Ca-MnOx
H
FE of HClO (%)
Calcination temperature (°C)
I
FE of HClO (%)
none
Mn-N
Mn-H
Mnganese salt using used for precursor solution

Figure S3. FEs for HClO generation on *MO_x*/FTO under dark conditions. Related to Figure 2.

The electric charge was 2 C (1000 s at a steady photocurrent of 2 mA) under various conditions. (A) FE (HClO) and FE(O_2) on *MO_x* (0.03 M)/FTO in 0.5 M aqueous NaCl solution. (B) FE (HClO) and apparent MnO_x-coverage calculated from XPS results for bare FTO and MnO_x/FTO photoanodes, which were prepared by coating the manganese precursor solution with different concentrations (0.01–0.1 M) to change the MnO_x loading amount. (C) FE (HClO) on bare FTO and *MO_x*/FTO in 0.005 M or 0.5 M aqueous NaCl solution. (D) FE (HClO) on MnO_x (0.1 M)/FTO in aqueous NaCl solution with various concentrations (0.005–2 M). (E) FE (HClO) on MnO_x (0.1 M)/FTO anode in 0.5 M aqueous NaCl solution with various pH (pH 3–9). (F) FE (HClO) on various *MO_x*/FTO anode at steady potentials (1.5–1.8 V vs. SHE, pH=7) in 0.5 M aqueous NaCl solution. pH was adjusted by using NaOH and HCl. For the *MO_x*/FTO electrodes (1.5 cm x 4 cm) used in the experiments of (B), (C) and (D), the concentrations of the coated metal precursor solution were 0.1 M for Mn, Co, Ni, and Fe, and 0.03 M for Rh. (G-i) XRD patterns of the various MnO_x/FTO anodes prepared by electrochemical deposition and followed by calcination at various temperatures. In the XRD pattern of MnO_x(ED-S(100°C)/FTO anode, no diffraction peak originated from the coating metal oxide, indicative of their amorphicity. (G-ii) FE (HClO) for MnO_x/FTO anodes prepared by electrochemical deposition followed by calcination at various temperatures, and that for the Ca-MnO_x anode in a 0.5 M aqueous NaCl solution. (H) FE (HClO) on MnO_x/FTO electrodes prepared by changing different calcination temperatures (300-550°C) in 0.5 M aqueous NaCl solution. (I) FE (HClO) on MnO_x/FTO electrodes prepared by using different manganese precursor salt ($Mn(NO_3)_2$•$6H_2O$ and manganese bis(2-ethylhexanoate)) The obtained electrodes are denoted that Mn-N, Mn-H, respectively.

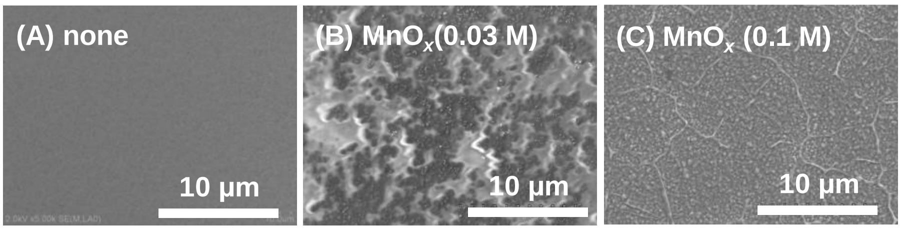

Apparent MnO_x coverage	0%	65%	99%
FE(HClO)	41.3%	< 2.4%	< 1.6%

Figure S4. SEM images and MnO_x coverages of (A) FTO substrate and (B)(C) MnO_x/FTO anodes. Related to Figure 3.

Manganese precursor solutions with different concentrations ((B) 0.03 M and (C) 0.1 M) were spin-coated on FTO, and calcined at 400 °C. The apparent MnO_x coverage was evaluated by using XPS spectra of Mn 2p to Sn 3d and these counts per seconds (cps) coefficients.

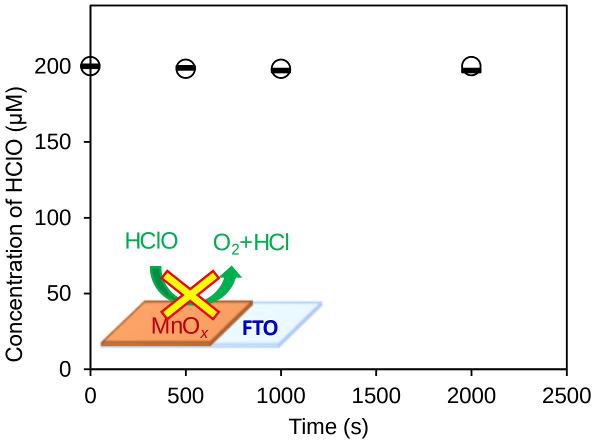

Figure S5. HClO degradation behavior for MnO_x (0.1 M)/FTO under dark conditions. Related to Figure 4, 5.
HClO degradation reactions were performed by using MnO_x (0.1 M)/FTO under dark conditions in 0.5 M aqueous NaCl solution (35 mL) with initial addition of 200 μM of NaClO; (i) without current flow (-), and (ii) at a steady current of 2 mA (○).

References:

Ramírez, A. et al. (2014) Evaluation of MnO_x, Mn_2O_3, and Mn_3O_4 Electrodeposited Films for the Oxygen Evolution Reaction of Water. *J. Phys. Chem. C 118*, 14073−14081.

Qi, Z., Younis, A., Chu, D., Li, S. (2016) A Facile and Template-Free One-Pot Synthesis of Mn_3O_4 Nanostructures as Electrochemical Supercapacitors. *Nano-Micro Lett. 8*, 165–173.

Iguchi, S., Miseki Y., Sayama, K. (2018) Efficient Hypochlorous Acid (HClO) Production *via* Photoelectrochemical Solar Energy Conversion Using a $BiVO_4$-based photoanode. *Sustainable Energy Fuels 2*, 155–162.
